# The *Mycobacterium avium* Complex: Genomics, Disease, and Beyond

**DOI:** 10.3390/microorganisms13102329

**Published:** 2025-10-09

**Authors:** Sofia Matos, Isabel Portugal, João Perdigão

**Affiliations:** Research Institute for Medicines (iMed.ULisboa), Faculdade de Farmácia, Universidade de Lisboa, 1649-003 Lisbon, Portugal; sfe.matos@campus.fct.unl.pt (S.M.); iportugal@ff.ulisboa.pt (I.P.)

**Keywords:** NTM, *Mycobacterium avium*, mycobacteriosis, population structure, diversity

## Abstract

Nontuberculous mycobacteria are opportunistic pathogens increasingly associated with human disease. Within this group, the *Mycobacterium avium* complex (MAC), which includes *M. avium*, *M. intracellulare* and *M. intracellulare* subsp. *chimaera*, is the most frequent cause of infection. The increase in MAC cases worldwide has made it crucial to understand their population structure, clinical relevance and resistance mechanisms. Recent advances in whole-genome sequencing (WGS) and molecular approaches have improved the knowledge of taxonomy, population structure and genetic diversity, while also enabling the investigation of transmission and epidemiology. Clinically, MAC most often causes chronic pulmonary disease, but extrapulmonary forms, including disseminated disease, also occur. Presentation can vary by infecting species, while host factors such as pre-existing lung disease or immunosuppression further increase the risk. Treatment outcomes remain less favourable than desired, in part due to antimicrobial resistance involving de novo-acquired mutations. Pathogenesis is also influenced by interactions between MAC and host cells, including mechanisms of immune evasion and inflammatory modulation. In addition, emerging evidence suggests that gut–lung axis dysbiosis may influence susceptibility to MAC infection. This review outlines current knowledge on the population structure, clinical significance, resistance and host–pathogen interactions of MAC.

## 1. Introduction

Nontuberculous mycobacteria (NTM) are becoming an increasing public health concern, with opportunistic infections on the rise [1]. In some regions, particularly developed countries, the incidence of NTM infection continues to increase while tuberculosis declines [2]. These organisms are ubiquitous in the environment, commonly found in the soil, water, and dust. Taxonomically, these microorganisms are traditionally grouped within the *Mycobacterium* genus, which includes additional prominent pathogens from a public health standpoint such as the *Mycobacterium tuberculosis* complex, *Mycobacterium leprae* and other NTM. However, recent phylogenomic analyses have challenged this taxonomy, with Gupta et al. proposing a division of the genus into five groups: *Mycobacterium*, *Mycolicibacterium*, *Mycolicibacillus*, *Mycolicibacter* and *Mycobacteroides* [3]. This split remains under discussion, with some authors recommending reconstitution of the original *Mycobacterium* genus [4], while others support retaining *Mycobacterium* and recognising *Mycobacteroides* [5]. NTM can also be broadly classified as slow-growing or rapidly growing species, based on their in vitro growth rates [6]. Rapid growers, such as the *Mycobacteroides abscessus* complex, often cause severe and difficult-to-treat pulmonary and skin infections, and form colonies within three to seven days [7]. On the other hand, slow growers require more than seven days to form visible colonies. The latter group includes the *Mycobacterium avium* complex (MAC), which is the most common cause of chronic pulmonary disease and disseminated infection among NTM [8]. The MAC stands out as a predominant cause of morbidity and mortality worldwide, with *M. avium*, *M. intracellulare* and *M. intracellulare* subsp. *chimaera* (*M. chimaera*) representing relevant pathogenic species.

The increasing number of NTM infections may reflect ongoing improvements in diagnostic methods, greater awareness of NTM and associated pathology, ageing and immunosuppression, as well as new invasive medical practices [9,10,11,12]. However, a significant proportion of prevalent cases are likely underreported due to diagnostic challenges and the lack of mandatory disease notification. As in many other European countries, MAC species are also the most frequently isolated NTM species associated with disease in Portugal.

The most common manifestation of MAC infection is pulmonary disease (PD), but it can also cause disseminated disease, endocarditis, lymphadenitis in children, osteomyelitis and skin and soft tissue infections [13]. These different disease types can be associated with different etiologic agents of this group. Typically, immunocompromised individuals are the most affected, but other risk factors, such as pre-existing lung disease, can also contribute to development of disease [14,15,16]. Older people are also at higher risk for developing MAC infection [17].

This review provides an overview of the population structure, clinical significance, resistance mechanisms, and host–pathogen interactions of MAC species, reflecting their growing importance in human disease.

## 2. Population Structure and Genetic Diversity

The first species assigned to the MAC were *M. avium* and *M. intracellulare*. Other species are assigned to the complex if they have sufficient similarity to the type strains of either of the original species. Advances in molecular techniques and genetic-based taxonomy have helped the allocation of some MAC variants to species of this complex, including *M. arosiense*, *M. bouchedurhonense*, *M. colombiense*, *M. lepraemurium*, *M. paraintracellulare*, *M. marseillense*, *M. timonense* and *M. vulneris* [18,19,20,21,22]. Recently, percentages of similarity to *M. avium* ATCC 25291 or *M. intracellulare* ATCC 13950 have been proposed to classify MAC species, using the full 16S rRNA sequence, the Telenti fragment of the *hsp65* gene, the Adekambi fragment of the *rpoB* gene, combined analyses of these three genes, or average nucleotide identity (ANI) values [19]. However, this group has kept on suffering constant taxonomic rearrangements, both regarding its species and subspecies, reflecting the underlying genetic diversity that complicates population structure analysis.

Although initially regarded as a rare infection in humans, *M. avium* gained more attention during the onset of the human immunodeficiency virus/acquired immunodeficiency syndrome (HIV/AIDS) pandemic in the 1980s, when cases of disseminated disease increased in individuals with low CD4+ T-cell counts (<50 CD4+ T-cells per mm^3^) [23]. This species is now divided into four genetically similar subspecies, but that vary in pathogenicity, host tropisms and ecological niches: *M. avium* subsp. *avium* (MAA) is the main cause of avian mycobacteriosis; *M. avium* subsp. *hominissuis* (MAH), an opportunistic pathogen that can infect humans, swine and other animals; *M. avium* subsp. *paratuberculosis* (MAP) is primarily associated with Johne’s disease in cattle and other ruminants; and *M. avium* subsp. *silvaticum* (MAS) mainly affects wood pigeons [24,25]. MAH is the most significant when it comes to human disease, but MAA has also been isolated from human specimens and MAP has been suggested as a possible cause for Crohn’s disease, although this association remains unproven [26]. These taxonomic divisions reflect genomic differences within *M. avium* subspecies, which are increasingly being clarified through molecular and genomic analyses.

Turenne et al. demonstrated that MAH displays the highest genetic diversity within the *M. avium* subspecies. The other subspecies have emerged from MAH through selective pressures into two separate groups: the “bird-type” group, comprising MAA and MAS, and the MAP group, likely through the accumulation of non-synonymous single nucleotide polymorphisms (SNPs) [27]. This pattern has also been observed in more recent phylogeny studies, including a global phylogenetic analysis of *M. avium* which showed the diversity of MAH strains when compared to the more clonal structure of MAP and MAA/MAS subspecies [28].

In contrast to *M. avium*, whose subspecies have long been recognised based on ecological and pathogenic differences, the taxonomy of *M. intracellulare* and closely related species has been subject to ongoing reclassification, particularly with the advent of whole-genome sequencing. Unlike *M. avium*, which is typically associated with some sort of immunosuppression, *M. intracellulare* is linked with pulmonary disease in immunocompetent people, particularly those with pre-existing lung disorders [14]. Initially, only these two species were recognised as distinct species of the MAC, alongside genetic variants MAC-A through H, based on the sequencing of the internal transcribed spacer (ITS) located between the 16S rRNA and 23S rRNA coding genes [29]. But, in 2004, Tortoli et al. proposed to elevate one of these variants, MAC-A, to the species rank, designating it *M. chimaera* [30]. Recent studies based on whole genome sequencing (WGS), ANI and DNA-DNA Hybridization (DDH), have reclassified *M. chimaera* as *M. intracellulare* subsp. *chimaera* and *Mycobacterium yongonense* as *M. intracellulare* subsp. *yongonense*. The latter has also been proposed to be fused into *M. intracellulare* subsp. *chimaera*. The same studies also revealed high ANI and DDH values between *M. intracellulare* and *M. paraintracellulare*, leading to recommendations of absorbing *M. paraintracellulare* into *M. intracellulare* subsp. *intracellulare* [31,32,33]. All these findings reflect the complexity of the taxonomic classification within the MAC.

Traditional typing of MAC isolates relied on serological methods or phenotypic characteristics, which often lacked the resolution to distinguish closely related species and subspecies. Historically, DNA-based methods have been used more frequently to type MAC isolates, including: pulse-field gel electrophoresis (PFGE), which separates DNA fragments by size using an alternating electric pulse; restriction fragment length polymorphisms (RFLP), which involves digesting PCR amplicons with restriction enzymes and comparing the resulting fragment sizes on an agarose gel; and random amplified polymorphic analysis (RAPD), which compares DNA fingerprints generated from randomly amplified DNA fragments. Other approaches, such as mycobacterial interspersed repetitive unit-variable number of tandem repeats (MIRU-VNTR) or *M. avium* tandem repeats-variable number of tandem repeats (MATR-VNTR), focus on quantifying the number of repeated sequences in specific genomic locations [34,35,36]. Genotyping is an important step which can help identify the likely source of infection and distinguish relapses from exogenous reinfections with new strains, which could be necessary to institute adequate treatment regimens [36]. In addition to these techniques, amplification of insertion sequences (IS) can be useful to identify subspecies of *M. avium* and the probable source of the isolate (for example, bird type or swine/human type). IS*900* is usually present in MAP isolates; IS*901* is more often associated with bird-type isolates; IS*1245* is commonly found in *M. avium* (especially MAH) isolates; and, IS*1311* is also frequently detected in *M. avium* isolates [37,38]. Since these insertion sequences are not found in *M. intracellulare*, detection of the DT1 region can also be used to distinguish this species from *M. avium* [39]. However, DT1 is also present in *M. chimaera*, and another region, the SR1, can be used to distinguish these isolates from other *M. intracellulare* isolates [40].

In addition to IS targeted screening, sequence-based identification methods have also played an important role in distinguishing species of the complex. Commonly sequenced regions include the 16S rRNA, *hsp65*, *rpoB*, and the ITS region [34,41]. More recently, WGS has become a valuable tool for differentiating species and subspecies of the MAC, enabling the detection of genetic diversity, which gives insights into population structure and can aid in clinical diagnosis, including identification of SNPs linked to drug resistance [33]. WGS is also useful for outbreak investigations, allowing the tracking of transmission chains and aiding in the establishment of epidemiological links [42]. However, a comprehensive population-level analysis of this complex is still lacking, often due to limited information, such as small sample sizes or restricted geographic coverage.

Nonetheless, recent large-scale WGS studies have significantly advanced the understanding in this field. One study analysed 1230 *M. avium* isolates across all subspecies, aiming to reconstruct global phylogeny, identify mutation hotspots and detect subspecies-specific genes. The resulting phylogenetic tree, aligned to a MAP reference genome, confirmed previous observations regarding *M. avium* population structure, including the extensive genetic diversity of MAH, displaying multiple clades, while MAP and MAA/MAS formed two distinct highly conserved clades. In fact, based on this structure, the authors suggested the division of MAH into further subspecies. Supporting this, the pangenome analysis revealed a smaller core genome and large pangenome for MAH, indicating high genomic plasticity that may reflect the need for adaptation, since they are ubiquitous in the environment. For MAH, lineage-specific features such as plasmid content and clade identity may also influence virulence or disease progression, as exemplified by strains related to strain TH135. In contrast, MAP had the smallest pangenome and largest core genome, while MAA/MAS appeared intermediate between the two [28].

Exploring genetic diversity and population structure can be helpful for understanding and predicting clinical outcomes and for overcoming diagnostic challenges, but it is also essential to determine sources of infection and transmission routes. Inhalation or ingestion of contaminated aerosols is the most probable transmission route, as MAC members grow and persist in water distribution systems, colonising showerheads and faucets, making them a likely source of infection [1]. Their natural resistance to disinfectants, such as chlorine, and their ability to survive in low pH can contribute to their persistence in these systems. This resistance is likely related to a rich lipidic membrane and the ability to form biofilms in these locations [43].

Beyond plumbing systems, MAC species can also persist in healthcare settings, where they may colonise medical equipment and contribute to nosocomial infections. For instance, mycobacteria can also colonise bronchoscopes, their filters and the water used to clean these, posing a risk of transmission during procedures [9].

One of the most significant and well-documented MAC outbreaks was later traced to contaminated medical equipment. In 2015, an outbreak of *M. chimaera* infections following open heart surgery was associated with heater-cooler units (HCU) manufactured by LivaNova (London, UK) [11,44]. Initial analysis using RAPD-PCR identified two patient clusters, prompting environmental sampling, and cultures from HCUs present in operating rooms and from all drinking fountains in the hospital were positive for *M. chimaera*. Subsequent investigations applied WGS to identify the source of infection in multiple outbreaks, showing high similarity between patient isolates and those recovered from LivaNova HCUs utilised during surgery, suggesting contamination of these devices at the production site [44].

WGS has also been applied to characterise MAC transmission in clinical settings. Another study, based on WGS of isolates collected from patients at respiratory clinics in a London hospital, aimed to characterise the population structure of MAC and determine transmission chains. MAA, MAH, *M. intracellulare* and *M. chimaera* isolates were grouped into lineages using fastBAPS, and global genomes were incorporated to infer possible transmission clusters, based on pairwise SNP distance. However, one key limitation of this study was the lack of environmental sampling, preventing identification of possible environmental sources of infection. In most cases, no clear epidemiological links between patients could be established, except for one *M. chimaera* cluster. Interestingly, isolates associated with HCUs were clustered together in a distinct lineage derived from lineages composed exclusively of human isolates. No other globally circulating lineages were identified [45].

Another large scale multi-national European study applied WGS to over 600 MAC isolates collected from patients in Germany, Switzerland and France to investigate transmission and population structure [46]. Using a 12 SNP threshold, the authors identified multiple clusters, but most appeared within the same hospital centres and lacked clear epidemiological links, making direct transmission unlikely. The broader phylogenetic analysis revealed mainly trans-European clusters across *M. avium*, *M. intracellulare* and *M. chimaera*, with a smaller number of transcontinental *M. avium* clusters. In addition, plasmid prediction analysis suggested that 98.7% of *M. chimaera* isolates carried plasmids, a high rate that is consistent with other reports [47], raising questions about their role in genomic diversity or adaptation [46].

MAC persistence in water systems may also involve interactions with free-living amoebas, which have been suggested as a protective niche for these organisms. This has been well documented for *Legionella pneumophila*, where intracellular survival is thought to drive an accidental evolution of traits that enhance virulence in humans. A similar mechanism has been proposed for MAC. Living inside amoebas could also be a way to improve their survival in water systems and favour the selection of traits associated with virulence, which has been observed multiple times in vitro [48,49]. In fact, the ability to persist within these amoebas may contribute to enhanced survival of NTM inside macrophages [50]. Genomic analyses further support the role of environmental adaptation in shaping MAC diversity. Keen et al. examined whether the genetic diversity of each *M. avium* isolate could be associated with its environmental origin. Their findings revealed higher similarity between pangenomes (and core genomes) of isolates from the same origin, suggesting that certain accessory genes in *M. avium* may contribute to adaptation to specific environments. Additionally, the authors found variation in virulence factors between MAC species, as well as in *M. avium* isolates from different sources [51].

WGS is a powerful tool for studying population structure and phylogenetic relationships within the MAC. By enabling comparative genomics, it has played a critical role in unveiling genomic diversity that might otherwise go unnoticed, and it has been essential for species and subspecies (re)classification [32]. Traditionally, WGS has been used primarily for research purposes and public health investigation, particularly for outbreak tracking and identifying transmission sources [42], but it is now gaining traction as a valuable diagnostic tool, particularly as sequencing becomes faster and more affordable [52]. One bioinformatics algorithm, developed and tested at the University of California, Los Angeles, showed promising results for clinical diagnostics, provided that high-quality genomes are available for comparison [53]. Having the complete genomic data can be useful for species identification, to establish epidemiological links through the genome-wide analysis of SNP distance between isolates, and even to predict antibiotic resistance through previously described resistance associated variants. More recently, the NTM-Profiler pipeline has been developed to identify NTM species from WGS data. It can also predict antimicrobial resistance in *M. leprae* and *M. abscessus*. Although still under active development, this tool has already been applied in MAC genomic studies [46], as well as other NTM studies [54], highlighting the importance of WGS in clinical and epidemiological contexts.

The applications of WGS are not only useful for surveillance and outbreak investigation, but may also play a role in understanding disease progression, treatment response and clinical outcomes.

## 3. Clinical Significance of MAC Infections

Employing WGS in diagnosis can be important to distinguish between MAC species, particularly because their clinical manifestations can be similar. While most MAC species are associated with chronic PD, certain clinical manifestations appear to be more frequently linked to specific species or subspecies (Table 1). PD, however, can typically have two different clinical presentations: fibrocavitary (FC) disease, in which cavitary lesions develop in the upper lobes of the lungs, and nodular bronchiectatic (NB) disease, which can be cavitary or non-cavitary and is characterised by the development of bronchiectasis in the right middle lobe and lingula with multiple nodules [55].

FC disease primarily affects older male smokers with underlying lung disease, such as chronic obstructive pulmonary disease (COPD), silicosis, cystic fibrosis (CF), chronic aspergillosis or a previous history of pneumonia or tuberculosis [56]. NB disease occurs more frequently in nonsmoking post-menopausal females with bronchiectasis but without any other pre-existing lung disease. These patients often present with distinct body features, such as being tall, thin and having chest anomalies, like pectus excavatum, mitral valve prolapse or scoliosis [57]. Previous studies show that cavitary disease is more associated with *M. intracellulare*, but the predominant species causing NB disease varies across studies [55,58,59]. Cavitary forms of the disease have a worse prognosis, faster progression and are more difficult to treat [55,60,61]. This may in part explain why *M. intracellulare* is often associated with more severe and advanced disease and lower treatment success rates [62].

*M. avium* complex pulmonary disease (MAC-PD) can also present as hypersensitivity pneumonitis, a form of lung inflammation associated with hot tub use [63].

**Figure 1:**
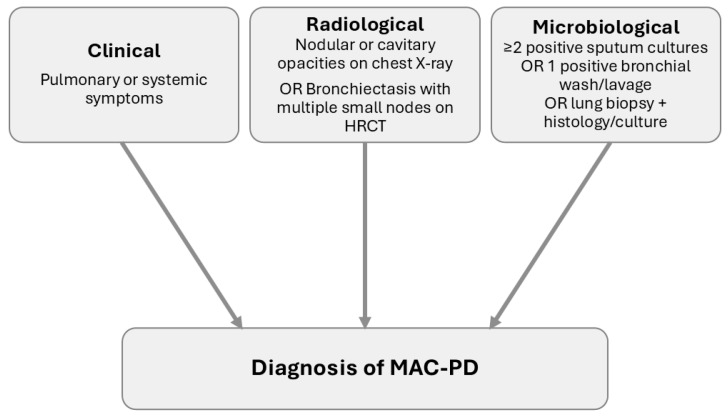
Schematic representation of the American Thoracic Society diagnostic criteria for *Mycobacterium avium* complex pulmonary disease (MAC-PD). Diagnosis requires all three criteria: (i) clinical symptoms, (ii) compatible radiological findings on chest X-ray or high-resolution computed tomography (HRCT), and (iii) microbiological confirmation through positive cultures or biopsy.

Diagnosing lung disease can be challenging, as isolation of MAC from respiratory specimens can simply indicate colonisation or contamination, rather than true infection causing disease. Furthermore, MAC-PD can have a nonspecific presentation, delaying diagnosis or leading to misdiagnosis. For this reason, the American Thoracic Society (ATS) has defined clinical, radiological and microbiological criteria for the diagnosis of MAC-PD (Figure 1). Clinically significant specimens must be isolated from a patient with pulmonary or systemic symptoms along with nodular or cavitary opacities on chest radiograph, or bronchiectasis with multiple small nodules on a high-resolution computed tomography (HRCT). Microbiologically, one of the following three criteria must apply: at least two positive sputum cultures; one positive bronchial wash or lavage culture; or a transbronchial or other lung biopsy with mycobacterial histological findings plus a positive culture from either the biopsy or sputum or bronchial washings [64].

Members of the complex are also responsible for a range of extrapulmonary diseases. The most frequent is lymphadenitis, which affects mostly healthy children and is most often caused by MAH [65]. Other extrapulmonary diseases include cutaneous infections, like papular nodular lesions, ulcerations, erythematous plaques and abscesses [66], or musculoskeletal infections, such as tenosynovitis, septic arthritis and osteomyelitis [67,68,69]. MAC is also a major cause of disseminated disease in individuals with advanced HIV/AIDS or with other forms of immunosuppression [23]. *M. avium* is the most frequently isolated species in these cases, particularly in AIDS (acquired immunodeficiency syndrome) patients, but *M. intracellulare*, *M. chimaera* and even *M. colombiense* have also been associated with disseminated disease in this population [70,71,72]. *M. chimaera* has also been connected to a distinct clinical manifestation, endocarditis following open-chest heart surgery, although this was associated with a specific outbreak event [11].

Although ATS guidelines recommend starting treatment when a patient meets diagnostic criteria, there is ongoing debate about whether this is always needed [73,74]. Meeting the criteria does not necessarily imply disease progression, and treatment can be lengthy and associated with possible side effects. However, there are some that may influence disease progression and therefore impact the decision to start treatment. These include low body mass index (BMI) and poor nutritional status, presence of cavitary lesions, extensive disease and a positive acid-fast bacilli smear [74].

The standard treatment for MAC infection recommended by the ATS does not vary between different species and focuses on PD [64]. However, the same treatment is used for other types of infection, as there are no other recommendations available [75,76]. This regimen consists of a combination of a macrolide (clarithromycin or azithromycin), ethambutol and a rifamycin, usually rifabutin or rifampin. For non-cavitary NB disease, the drugs should be taken three times per week. For cavitary forms and other severe cases, the drugs should be taken daily and a parenteral aminoglycoside should be added, normally amikacin or streptomycin. It is recommended to continue treatment for at least twelve months after culture conversion [64].

Despite the effectiveness of macrolide-based regimens compared to previously used regimens, these treatments are long and can entail multiple side effects, like gastrointestinal (GI), cutaneous, hepatic, visual or auditory issues [77]. When resistance or intolerance to first-line drugs occurs, alternatives such as clofazimine, fluoroquinolones, linezolid and bedaquiline may be considered. However, the efficacy of these treatments remains uncertain and, in some cases, can lead to macrolide resistance, if used in the setting of intolerance. In cases of resistance, combining surgery with aminoglycoside therapy is also a potential solution [64].

Although treatment improved after the introduction of macrolide-based therapy, treatment outcomes are still not as favourable as desired, with many patients failing to achieve culture conversion. For example, a study evaluating outcomes based on disease severity showed culture conversion rates of 85% for mild disease, compared to just 61% in those with severe disease [61]. For severe disease, time until a favourable outcome was also longer than for mild disease. Outcomes also differ according to radiological phenotype, even within cavitary forms: conversion rates of 81% and 60% have been reported for cavitary NB and FC disease, respectively [78]. By contrast, much poorer results have also been observed, with culture conversion achieved in only 31% of patients, although only 69% received guideline-based therapy [79]. Outcomes can also vary by infecting species, with favourable microbiological responses in 74% of *M. avium* cases compared to just 56% in *M. intracellulare* [62]. Prolonged treatment regimens and significant side effects highlight the need for continued research into more effective and tolerable therapies, particularly in severe cavitary forms where poor outcomes and resistance remain a challenge [74].

Antimicrobial resistance is a major challenge in the management of MAC infections and is strongly associated with poor treatment outcomes. Because macrolides are the most effective drug against MAC infections, resistance to this class is a major factor contributing to poor treatment outcomes. Patients with resistant strains given alternative regimens show extremely low culture conversion rates, ranging from as low as 14% to 40% [80,81], and increased mortality compared to those with susceptible strains [82]. Relapse also occurs in MAC infections and may be associated with the development of macrolide resistance in some strains, especially when the triple drug regimen is not applied [83]. Resistance to macrolides is associated with inadequate treatment regimens, particularly prior macrolide monotherapy or the combination of a macrolide and a fluoroquinolone [83], which select for mutations in the peptidyltransferase region of the 23S rRNA gene. Multi-drug regimens without ethambutol may also be a cause for the development of resistance, and this drug may sometimes be excluded from the treatment regime due to its side effects [82]. Other factors such as cavitary disease and high bacterial burden have also been linked to worse outcomes and may increase the likelihood of resistance [82,84]. Previously described mutations include substitutions at positions A2058 and A2059, which confer high-level resistance to macrolides [85,86,87]. Resistance is uncommon in patients with no history of treatment, but can develop under inadequate therapy, severely limiting treatment options.

Aminoglycoside resistance, although less frequent, is most commonly associated with the A1408G mutation in the 16S rRNA gene [88]. Other mutations have been associated with resistance to aminoglycosides in other mycobacteria [89], but have been less frequently encountered in MAC isolates [90]. Although most isolates remain susceptible, there have been reports of resistant *M. avium* and *M. intracellulare* strains, usually detected in patients previously exposed to aminoglycoside treatment [90,91]. While aminoglycoside-modifying enzymes contribute to resistance in some mycobacterial species, they do not appear to play a significant role in MAC [92]. The introduction of liposomal inhaled amikacin (ALIS) may be more effective when compared to free amikacin, as it delivers the drug directly to the lungs. For patients with macrolide resistant strains, the addition of ALIS to guideline-based treatment increased culture conversion rates, when compared to patients on only the three drug regimen [80].

For other drugs commonly used for MAC treatment, such as rifamycins and fluoroquinolones, the resistance mechanisms remain unclear. While rifamycins show in vitro activity, their clinical efficacy is limited. Although mutations in *rpoB* have been reported [93], clinical significance of these is still uncertain [94]. Similarly, mutations in *gyrA* or *gyrB* have been described [95], but there seems to be a lack of correlation with fluoroquinolone resistance [96]. Other agents, such as clofazimine, linezolid and bedaquiline have shown activity against MAC and are increasingly considered for second line treatment, but their resistance mechanisms have not been studied [97,98,99].

However, drug susceptibility testing in MAC remains challenging, as reproducibility across laboratories is limited and clinical breakpoints are not well established, complicating the interpretation of in vitro testing [100]. For most drugs used against MAC, in vitro testing has limited correlation with clinical outcomes, with the exception of macrolides and amikacin [101], for which resistance is associated with treatment failure. Although recent guidelines recommend breakpoints for some of the drugs, including macrolides and aminoglycosides, there is still an effort ongoing to establish these breakpoints with more certainty [100].

In addition to acquired mutational resistance, MAC are inherently difficult to treat due to phenotypic mechanisms that reduce antibiotic efficacy. One important mechanism is their ability to survive inside macrophages, where drug penetration and altered phagosomal conditions reduce antimicrobial activity [102]. Another factor complicating treatment is growth in biofilms. MAC forms biofilms in water distribution systems and medical devices [9,103], which reduces permeability and increases tolerance to antimicrobials and disinfectants [43]. Efflux pumps may also play a role in resistance, with previous reports showing overexpression of MAV_1406 and MAV_1695, pumps associated with macrolide efflux, as well as reduced minimum inhibitory concentrations (MICs) upon inhibition of the efflux pumps [104]. This might suggest a contribution to clinical resistance. Taken together, resistance in MAC involves both mutational and phenotypic mechanisms, many of which remain poorly defined, and it continues to be a major barrier to effective treatment and successful outcomes.

Potential associations between strain or lineage and disease severity have been suggested, although evidence remains limited. Disease progression has been linked to MAH strain TH135, associated with progressive PD. This strain includes plasmid pMAH135, which carries genes that may be related to disease progression [105]. Another study described a highly virulent *M. intracellulare* strain that caused severe PD in an immunocompetent host and demonstrated increased growth and survival in both THP-1 macrophages and mice compared to other clinical isolates [106]. However, it is uncommon for in vitro and in vivo findings to align with clinical outcomes as in this case, highlighting the importance of host factors in disease progression and severity [106].

## 4. Host–Pathogen Relationship

MAC species initiate infection by attaching and invading the mucosa of the respiratory tract or the GI tract, after inhalation of aerosols or oral ingestion, respectively [1,107].

GI infection usually happens in immunocompromised individuals, particularly human immunodeficiency virus (HIV)-positive individuals [107]. Due to either its cell wall or the synthesis of glutamate, *M. avium* survives the acidic pH of the stomach and reaches the intestine [108], where it interacts primarily with enterocytes through receptors in the apical membrane and resides inside intracytoplasmic vacuoles [109]. Some of the genes involved in enterocyte invasion are absent in *M. intracellulare*, making it less effective than *M. avium* in the invasion of intestinal epithelial cells, highlighting its primary role as a lung pathogen [110]. Although the exact process remains unknown, it is likely that *M. avium* exiting the enterocytes to the mesenteric lymphatic nodes is the precursor for disseminated disease [111].

A similar process occurs during the invasion of respiratory tract epithelial cells, in immunocompetent patients. Although less is known about this pathway, there is the possibility of biofilm development for colonisation and infection of the bronchial mucosa [112]. In the alveolar space, the mycobacteria contact with and invade alveolar cells, having the ability to replicate in A549 human type II alveolar cells [113]. It has also been shown that MAC species bind to fibronectin in the extracellular matrix (ECM), adhering to epithelial cells. The ECM is only exposed in zones of epithelial damage, which explains the predisposition of individuals with underlying lung diseases for MAC-PD [114].

Neutrophils are part of the innate immune response, acting in the initial phase of MAC infection. Although neutrophil involvement is not very frequent, some of these immune cells are capable of phagocytosis and killing the mycobacteria [115]. Infected neutrophils can also produce tumour necrosis factor-α (TNF-α) and interleukin-12 (IL-12), stimulating nearby macrophages [116], the primary immune cells responsible for clearing the mycobacterial infection.

Adhesins present on the surface of mycobacterial cells mediate their interaction with epithelial cells. These include heparin-binding hemagglutinin and laminin-binding proteins, which are highly conserved across the MAC [117]. The epithelial cells uptake the mycobacteria through receptors associated with the cell cytoskeleton, implying its rearrangement after activation of small GTPase Rho [118]. There is also evidence suggesting that tyrosine phosphorylation is needed for the mycobacterial uptake, as inhibition of kinases decreases *M. avium* internalisation [119]. In both pathways, production of interleukin-8 (IL-8) is suppressed after the bacteria enters the cell, as well as regulated upon activation, normal T-cell expressed and secreted (RANTES) in the intestinal epithelial cells, and monocyte-chemotactic protein-1 (MCP-1) in the respiratory mucosa [120,121], resulting in decreased recruitment of immune cells and facilitating their survival and persistence.

Moreover, MAC species can evade the antimicrobial peptides (AMPs) secreted by the mucosal surfaces. AMPs are peptides secreted by the mucosa or delivered to infected phagocytic cells and may have direct antimicrobial activity or other modes of action, such as chemotaxis, inflammation and immune modulation and, wound and tissue repair. AMPs secreted by the intestine include α- and β-defensins, C-type lectin and cathelicidin that form pores in the membrane of the pathogen [122,123]. In the respiratory tract, there is secretion of defensins and cathelicidin [124]. Given the resistance of *M. avium* to AMPs, and since Polymyxin B can be used as substitute for these, one study aimed to screen for mutations that could be related to this resistance. The authors discovered that mutations in genes involved in cell wall composition, synthesis and permeability influence susceptibility to Polymyxin B, indicating that cell wall composition and related pathways are essential for resistance to AMPs [125].

MAC members contain serovar-specific glycopeptidolipids (ssGPLs) in the surface of the cell, which can be used to type the bacteria into serovars [126]. These glycolipids are similar in structure to non-specific glycopeptidolipids, which are composed of a lipopeptide core structure and attached oligosaccharide residues, which can have modifications. Depending on the carbohydrate attached, the ssGPL is going to be different [127], thus allowing the typing into serovars through serology. Initially, it was thought that certain serovars more frequently isolated from people with AIDS were specific to these patients [128]. However, it was later discovered that the same MAC serovar was isolated from people with AIDS and people without it, suggesting a geographic distribution or ecological factors as the cause for the frequency of isolation of some serovars [128]. While ssGPLs modulate a pro-inflammatory immune response, they can also promote immune tolerance, leading to less efficient immune response.

After the invasion of epithelial cells, the mycobacteria are internalised by phagocytic cells, mostly macrophages or monocytes. They can enter through a myriad of receptors, including complement receptors, mannose receptors, type-A scavenger receptors, Fcγ receptors, fibronectin and vitronectin receptors [110], and persist inside phagosomes that do not acidify and do not fuse with lysosomes, replicating and disseminating the infection to other macrophages. Phagosome and lysosome fusion can be inhibited by the binding of the oligosaccharide portion of ssGPLs to mannose receptors [129].

ssGPLs of *M. avium* bind to and stimulate toll-like receptor (TLR) 2 of the macrophage, resulting in the increase in mycobactericidal activity by activation of mitogen activated protein kinases, and consequent activation of nuclear factor-κB (NF-κB), involved in immune response regulation [130,131]. When this transcription factor is activated, there is an increase in the secretion of pro-inflammatory cytokines to promote phagolysosome fusion. The phagolysosome results from the fusion of a phagosome with a lysosome and has an important role in combating pathogens, presenting an acidic pH, degradation enzymes and reactive oxygen species [132]. However, *M. avium* has the ability to downregulate NF-κB overtime, inhibiting this fusion and thus surviving inside the phagosome [132].

The mycobacteria can assume an intracellular phenotype, which facilitates the infection of other macrophages and intracellular survival. When expressing this phenotype, the mycobacterium becomes more virulent and enters a new macrophage through receptors other than complement receptors, including scavenger receptors [133]. As there is no neutrophil activity after two weeks of infection [116], it is likely that the mycobacteria have started expressing an intracellular phenotype at that time.

**Figure 2:**
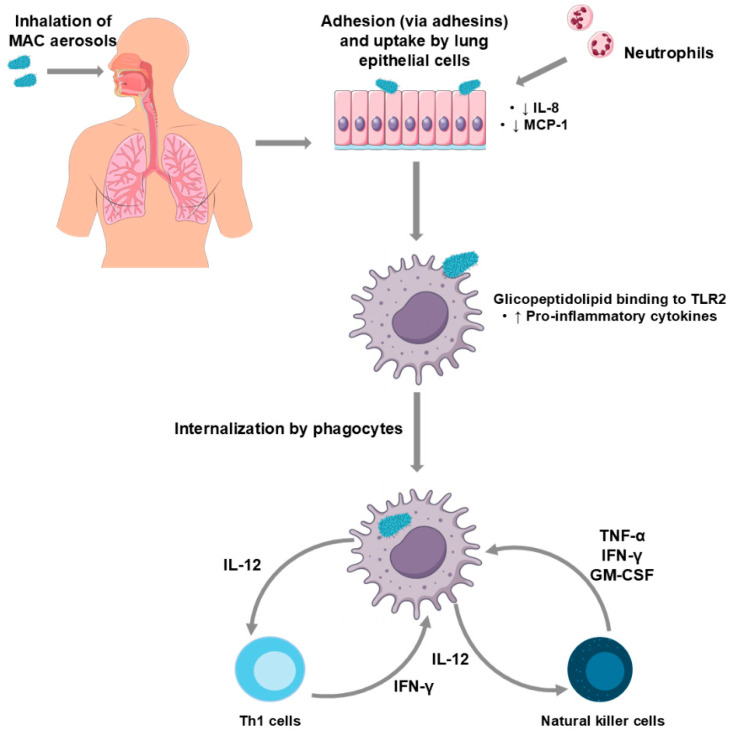
Schematic representation of the immunopathogenesis of *Mycobacterium avium* complex pulmonary disease (MAC-PD). Inhaled MAC organisms adhere to and are internalised by lung epithelial cells, leading to neutrophil recruitment and decreased interleukin-8 (IL-8) and monocyte-chemotactic protein 1 (MCP-1). Following phagocytosis, mycobacterial glycolipid binding to toll-like receptor (TLR) 2 triggers increased pro-inflammatory cytokine production. Infected phagocytes secrete interleukin-12 (IL-12), which activates T helper 1 (Th1) and natural killer (NK) cells. These cells, in turn, produce interferon-γ (IFN-γ), tumour necrosis factor-α (TNF-α), and granulocyte–macrophage colony-stimulating factor (GM-CSF), amplifying the immune response.

Natural killer (NK) cells are also part of the innate immune response against MAC pathogens, early in the course of infection. Infected macrophages produce IL-12, which stimulates NK cells to produce TNF-α, interferon-γ (IFN-γ) and granulocyte-macrophage colony stimulating factor (GM-CSF) that induce macrophage activity against the mycobacteria, inhibiting their growth [134]. IL-12 and TNF-α act synergistically to increase antimycobacterial activity in macrophages (Figure 2).

While the innate immune response plays a significant role in combating MAC infection, the adaptive immune response is also crucial. CD4+ T-cells are essential for infection control and, seemingly, CD8+ cells do not contribute as much to the adaptive immune response. CD4+ cells influence granuloma formation, although it still happens in the absence of these cells [135]. These cells differentiate into Th1 cells after contact with infected phagocytes, leading to the production of IFN-γ, which activates nitric oxide (NO) production in macrophages, killing mycobacteria. IL-12 is also produced, stimulating innate immune cells and inducing Th1 differentiation [136]. Th17 cells also have a relevant role against mycobacteria, recruiting neutrophils and aiding granuloma formation [136].

As NTM are ubiquitous in the environment, it is expected that contact with these mycobacteria is frequent. However, cases of NTM infection are sparse, implying that there are some factors in the host associated with developing disease. Another piece of evidence that suggests a role of host factors in developing MAC disease is that the percentage of patients that suffer from relapse with the same strain is similar to that of reinfection with another strain, which should not be the case when considering the rarity of NTM and MAC infections [137].

Some individuals are genetically predisposed to MAC infections due to mutations or polymorphisms in immune related genes. The Solute Carrier Family 11A member 1 (*SLC11A1*) gene encodes a transmembrane protein localised in late endosomes and lysosomes of phagocytic cells, involved in the transport of divalent cations. This protein influences the presence of metals and pH of the environment, NO production and modulates immune response [138]. Certain *SLC11A1* and promoter polymorphisms are associated with intracellular infection, including MAC disease. Loss of function or diminished expression is connected to higher susceptibility to infection [139].

NTM pulmonary infection presents very often in CF patients, with *M. abscessus* and MAC accounting for the majority of these infections. CF is an autosomal recessive disease due to variations in the cystic fibrosis transmembrane conductance regulator (*CFTR*), affecting the respiratory and GI tract and impacting metabolism and fertility in males [140]. In the respiratory tract, CF causes chronic inflammation and deficient clearance of secretions, creating the ideal environment for the development of pulmonary infections and bronchiectasis [140], including MAC colonisation and infection. Additionally, a relation between certain *CFTR* variations, especially Q1352H, and NTM pulmonary infection [141] has been reported. Prolonged use of macrolides can often be needed to manage CF, which can possibly lead to difficult-to-treat, macrolide-resistant NTM [142].

COPD is a chronic respiratory disease associated with a history of tobacco smoking. In 2021, it was the fourth leading cause of death worldwide [143]. This disease is characterised by emphysema and chronic bronchitis, leading to airway inflammation, lung tissue damage and accumulation of mucus in the airways, which creates favourable conditions for bacterial colonisation, including MAC species [144]. In addition, weakened immune defences and the use of inhaled corticosteroids may further increase susceptibility to MAC-PD in these patients [144,145].

Other genetic disorders can also be a risk factor for NTM infection. Primary ciliary dyskinesia, alpha-1-antitrypsin deficiency, tracheobronchomegaly, pulmonary alveolar proteinosis, common variable immunodeficiency, low body fat due to leptin deficiency and mutations in the macrophage-stimulanting-1 receptor gene can predispose to nontuberculous mycobacteria pulmonary disease (NTM-PD). Chronic granulomatous disease and auto-anti-IFN-γ antibodies can lead to higher susceptibility to other forms of NTM disease [10].

Mendelian susceptibility to mycobacterial disease (MSMD) is a group of genetic disorders that causes abnormal susceptibility to weakly violent mycobacterial infections, including the Bacillus Calmette-Guérin vaccine [146]. MSMD varies in severity and can be caused by multiple genetic errors, affecting different immunity pathways, particularly IFN-γ signalling [147].

In addition to these comorbidities, the gut-lung axis may also influence susceptibility to NTM-PD. The microbiota composition in one organ can affect the other, and changes/imbalances can contribute to the development or progression of certain diseases. For example, PDs like CF, asthma and COPD often display intestinal manifestations and are influenced by the composition of both the gut and lung microbiota [148,149,150,151]. The bidirectional relationship between gut and lung microbiota has also been increasingly studied in NTM-PD. Healthy lung microbiota is usually rich in *Prevotella*, *Streptococcus*, *Veillonella* and other species [152,153], while in individuals with NTM-PD, the overall diversity is not significantly different, but certain taxa such as *Prevotella* and *Veillonella* are altered [154]. The gastrointestinal microbiota is highly diverse, and dysbiosis has been linked to altered immune responses in the lung [155]. Lin et al. reported reduced alpha and beta diversity in the gut microbiota of NTM-PD patients, with lower abundance of *Prevotella copri*, which correlated with decreased TLR2 activation and greater susceptibility to disease [156].

MAC infection, especially *M. avium*, is a known comorbidity of HIV infection, especially in advanced stages. Even though this infection gained attention for being the most common opportunistic bacterial infection related to late-stage AIDS, the introduction of highly active antiretroviral therapy has helped decrease the number of cases in these patients [157]. However, it is still a relevant complication of AIDS, especially in cases of intolerance or resistance to the treatment regimen [158]. Other factors that decrease immunity also predispose to MAC infection, including immunosuppressive therapies for autoimmune disorders or post-transplant, biologics and chemotherapy [10].

## 5. Conclusions

The MAC represents a group of opportunistic pathogens with emerging clinical significance, due to both their ubiquitous nature and the increase in immunosuppressed individuals, as well as a longer life expectancy, which are both risk factors for developing MAC infection. The advances in molecular genomics and taxonomy have been crucial to classify the species within the group. This classification has implications in diagnosis, treatment and management of different MAC infections, but continues to be complex and in constant evolution, as the members of the complex are genetically very closely related.

Understanding the population structure of MAC has become increasingly important, not only to resolve its taxonomy, but also to recognise transmission patterns, identify possible reservoirs, and to associate particular strains with disease outcomes. WGS has played a key role in unveiling the genetic diversity within the complex, especially within MAH, and how certain strains may adapt to specific environments or display heightened virulence.

Diagnosis and treatment of MAC infections are notoriously difficult. Antimicrobial resistance, particularly to macrolides, poses one of the greatest challenges, as it undermines the effectiveness of the most important drug class against MAC disease. Amikacin is generally reserved for severe disease, but resistance can also emerge, and mechanisms for many other drugs are still poorly understood. In addition, susceptibility testing is not standardised, reducing its clinical utility. Together, these factors highlight the urgent need for improved understanding of resistance pathways and the development of shorter, more effective therapeutic strategies.

Understanding the pathogenesis of MAC species is also important for this research, including the different diseases caused by the different species. These mycobacteria invade the epithelial cells through adhesin-mediated binding and evade immune response by inhibiting phagolysosome fusion, surviving within the macrophage.

Host factors should also be considered when evaluating MAC infection. Multiple genetic predispositions or acquired immunodeficiencies can increase susceptibility to disease. Dysbiosis of the gut-lung axis is also linked to susceptibility to MAC disease.

The recent rise in MAC infections presents a growing public health concern and it is important to tackle it by researching genetic profiles, developing novel therapeutic strategies and raising awareness for these diseases in both healthcare environment and in the general public. However, current knowledge still has important limitations. Most genomic studies remain geographically restricted, resistance mechanisms remain unknown for most drugs, and experimental models often fail to capture the complexity of human disease. Future work should aim to expand global genomic datasets, clarify resistance pathways, and develop therapies that are both more effective and better tolerated, thereby paving the way to improved outcomes for patients affected by MAC infections.

## Figures and Tables

**Table 1:** Clinical manifestations associated with species and subspecies of the *Mycobacterium avium* complex (MAC).

Species	Clinical Manifestations
*Mycobacterium avium* subsp. *hominissuis*	Disseminated disease in
	immunocompromised individuals
	Pulmonary disease
	Lymphadenitis
*Mycobacterium intracellulare*	Pulmonary disease in immunocompetent individuals
*Mycobacterium intracellulare* subsp. *chimaera*	Endocarditis
	Pulmonary disease

## Data Availability

No new data were created or analysed in this study. Data sharing is not applicable to this article.
